# Medication access barriers and perceived health consequences in post-war Syria: a cross-sectional study in Homs

**DOI:** 10.1080/16549716.2026.2726661

**Published:** 2026-09-04

**Authors:** Rawan Al-Deeb, Ahmad Bishr Nasra, Mohammad Alaa Aldakak, Bayan Muhrat, Shahd Almansour, Ibrahim Auf, Hussein Almohammad, Fater Naimah, Ahmad Hajjo, Nawal Al-Sebai, Mohammad Keshe

**Affiliations:** a Faculty of Medicine, Homs University, Homs, Syrian Arab Republic; b Faculty of Medicine, Damascus University, Damascus, Syrian Arab Republic; c Faculty of Pharmacy, Homs University, Homs, Syrian Arab Republic; d Department of Chemistry, Faculty of Science, Homs University, Homs, Syrian Arab Republic

**Keywords:** Essential medicines, pharmaceutical supply chains, healthcare affordability, health systems recovery, conflict-affected populations

## Abstract

**Background:**

Armed conflict can disrupt medication access through damaged infrastructure, supply shortages, service disruption, transport barriers, and financial hardship. Community-level evidence remains limited in post-war Syria.

**Objective:**

To assess medication accessibility, perceived barriers and consequences, and factors independently associated with greater barriers among residents of Homs.

**Methods:**

A cross-sectional study using convenience sampling included 1,042 participants in Homs between 29 March and 22 April 2026. Data were collected using an adapted, Arabic-translated, pilot-tested questionnaire. A seven-item medication access barrier score was calculated (range, 7–35). Descriptive analyses, nonparametric bivariate tests, and multivariable linear regression with robust standard errors were performed.

**Results:**

Median age was 26 years (IQR, 22–42), and 62.0% were female. Self-reported poor economic status increased from 10.8% before the war to 30.3% during the war, and only 10.9% reported easy medication access during the war. The median barrier score was 22 (IQR, 20–26). High medication prices were the most frequently endorsed barrier (69.7%). Perceived consequences included health deterioration due to inability to access medications (68.5%) and deaths attributed to medication shortages (53.2%). In adjusted analysis, self-reported poor wartime economic status, student status, and greater distance to the nearest medication provider were associated with higher barrier scores, whereas less than high-school education was associated with lower scores.

**Conclusions:**

Medication access problems were commonly reported and were associated with socioeconomic and geographic factors. These findings may inform post-conflict strategies addressing medicine affordability, supply continuity, and access to care.

## Paper Context

**Main findings**: Medication access barriers were commonly reported in Homs, with high prices, service disruption, transportation and fuel shortages, and power-related medication damage prominent; self-reported poor wartime economic status, student status, and greater provider distance were associated with higher barrier scores.

**Added knowledge**: This study provides community-level evidence from post-war Syria on residents’ experiences of medication access barriers and perceived health consequences after prolonged conflict.

**Global health impact for policy and action**: The findings suggest that recovery planning in conflict-affected settings may need to combine reliable medicine supply, financial protection, transportation-sensitive services, and infrastructure resilience to support treatment continuity.

## Background

Access to healthcare and essential medicines is fundamental to human survival and constitutes a universal human right to be guaranteed without discrimination [1,2]. Armed conflict can severely undermine this right by damaging health facilities, disrupting pharmaceutical supply chains, displacing healthcare workers, restricting population movement, and reducing the availability and affordability of essential medicines [3–8]. These disruptions are particularly consequential for people with chronic diseases who depend on continuous treatment and regular clinical follow-up, as interruptions in medication access may contribute to disease progression, complications, hospitalization, and reduced quality of life [9].

Syria’s prolonged conflict has caused extensive damage to civil and health infrastructure, substantial population displacement, and severe losses within the healthcare workforce [10,11]. Medication access has been further affected by economic deterioration, trade restrictions, sanctions, and instability affecting the importation of medicines and pharmaceutical raw materials [12]. Recent evidence from Northern Syria has also demonstrated limited availability of essential medicines for noncommunicable diseases, reflecting the combined effects of disrupted supply chains, weak regulation, damaged infrastructure, and financial instability [7]. Similar patterns have been reported in other conflict-affected settings, including Sudan and Gaza, where medicine shortages, damaged facilities, transport constraints, and health-service disruption have substantially restricted access to care [13,14].

These challenges have persisted alongside severe humanitarian and socioeconomic pressures. The World Health Organization’s 2025 Health Emergency Appeal reported that almost 15 million people in Syria needed urgent healthcare assistance in 2024, with this number expected to rise to 16.7 million in 2025 [15]. The United Nations Office for the Coordination of Humanitarian Affairs also reported that 90% of Syrians were living below the poverty line, forcing many households to reduce spending on essentials such as food, water, healthcare, and education [16]. Disruption in the supply of medicines and medical equipment remains critical, particularly for essential medicines used for chronic conditions, including noncommunicable diseases [16].

Despite this growing evidence, community-level data from within post-war Syria remain scarce. Available research has largely focused on health-system disruption or medicine availability, with limited evidence describing how residents experience affordability barriers, geographic and transport constraints, service disruption, and the perceived consequences of limited medication access. To our knowledge, community-level evidence from Homs describing residents’ experiences of medication access barriers and their perceived consequences remains limited. Therefore, this study aimed to assess medication accessibility, current barriers, perceived health consequences, and factors independently associated with greater medication access barriers among residents of Homs during and after the war.

## Methods

### Study design

This quantitative cross-sectional study examined medication accessibility and its perceived health consequences among residents of Homs during and after the war between 29 March and 22 April 2026. Homs is a major city in central Syria whose residents experienced prolonged conflict-related disruption of healthcare and other essential services. The study followed the Strengthening the Reporting of Observational Studies in Epidemiology (STROBE) guidelines to ensure transparency and methodological quality.

A convenience sampling strategy was used. For recruitment planning, the minimum sample size was estimated using a single-population proportion formula, assuming an expected proportion of 50%, a 95% confidence level, and a 5% margin of error. This yielded a planning target of approximately 384 participants. Because convenience sampling was used, these assumptions were used only to guide recruitment and should not be interpreted as providing a 5% margin of error or population-level precision for residents of Homs. Recruitment continued throughout the planned data-collection period, resulting in a final sample substantially larger than the planning target.

The questionnaire was distributed both online and offline. Online distribution was carried out through social media platforms and local community groups involving residents of Homs city. To reach individuals with limited or no internet access due to the long-term impact of conflict, printed questionnaires were also distributed. Trained members of the research team recruited participants and distributed printed questionnaires mainly in hospitals and public places. Potentially eligible individuals were approached consecutively where feasible, although recruitment ultimately remained based on convenience and willingness to participate.

In some cases, face-to-face interviews were conducted with participants who were unable to read or had visual impairments, ensuring their inclusion in the study. Trained researchers administered these questionnaires using a standardized procedure, reading each question and response option exactly as written and recording the participant’s selected response without interpretation or prompting. The study information and consent statement were read aloud, and verbal informed consent was obtained before interviewer-administered participation. The mode of questionnaire completion was not retained as a separate variable in the final dataset; therefore, the numbers completing the questionnaire online, on paper, or through interviewer administration could not be reported separately.

### Inclusion and exclusion criteria

The inclusion criteria comprised Syrian individuals aged 18 years or older who lived in Homs city, had lived through the conflict period, and consented to participate. For online participants, mandatory screening questions confirmed age, residence in Homs, experience of the conflict period, and consent before access to the remainder of the questionnaire. The exclusion criteria included individuals under the age of 18, those residing outside Homs city, those who refused to participate, and submissions that could not be reliably analyzed because of substantial missing data or clearly contradictory response patterns across related questionnaire items. Before analysis, all submissions were reviewed for completeness and internal consistency during data cleaning. The online questionnaire did not technically restrict participants to one submission; therefore, potential duplicate responses were assessed after data collection by reviewing submission timestamps and exact response patterns.

### Measurement tools

The data collection tool was adapted from a previously published cross-sectional study that assessed the impact of war on medication accessibility and its health-related consequences among conflict-affected Sudanese citizens [6].

The original questionnaire was developed based on the study objectives and a review of relevant literature, then pre-tested for clarity and face validity. It consisted of three main domains: demographic and general characteristics, medication accessibility evaluation, and consequences of limited medication accessibility. In the original study, the medication accessibility evaluation subscale demonstrated acceptable internal consistency, with a Cronbach’s alpha of 0.741, while the medication accessibility consequences subscale showed good internal consistency, with a Cronbach’s alpha of 0.804.

For the present study, the questionnaire was adapted to the Syrian context and modified to reflect medication access challenges in Homs during and after the war. The original English-language questionnaire was translated into Arabic by members of the research team and reviewed by other team members for linguistic accuracy, clarity, and contextual appropriateness. Adaptations were limited to minor wording and contextual modifications and did not alter the number of items, response options, or scoring structure.

Before the main data collection, the adapted questionnaire was pilot-tested among 82 participants to assess clarity, feasibility, and internal consistency. Reliability analysis showed acceptable internal consistency for the seven-item medication access barriers subscale, with a Cronbach’s alpha of 0.788. The five-item consequences of limited medication access subscale showed moderate internal consistency, with a Cronbach’s alpha of 0.667. Because the five consequence items represented distinct perceived consequences rather than a single homogeneous construct, they were analyzed individually, and no composite consequence score was calculated. Only minor wording and contextual refinements were made following pilot testing, limited to a small number of demographic and contextual questions; none of the seven medication access barrier items, their response options, or their scoring were modified. Therefore, the pilot participants were retained in the final analytical dataset. Based on the pilot testing, the questionnaire was considered suitable for use in the main study.

The final tool included items on sociodemographic characteristics, residence and housing status before, during, and after the war, economic and employment status, chronic diseases, medication availability, distance to the nearest medication provider, current barriers to medication access, and perceived consequences of limited medication access. Medication access barriers and consequences were assessed using five-point Likert-scale items ranging from ‘strongly disagree’ to ‘strongly agree.’ The seven medication access barrier items were scored from 1 to 5 and summed to produce a possible score ranging from 7 to 35, with higher scores indicating greater perceived barriers. No predefined thresholds were available for categorizing the score as low, moderate, or high. Internal consistency of the seven-item score in the main sample was assessed using Cronbach’s alpha, corrected item-total correlations, and Cronbach’s alpha if each item was deleted. The five consequence items were analyzed individually and were not combined into a composite score.

Chronic diseases were self-reported, and participants could report more than one condition. Economic status before and during the war was based on participants’ subjective self-classification as poor, below average, average, or above average; no income-based thresholds were applied. Distance to the nearest medication provider was self-estimated by participants in meters. Residence before, during, and after the war referred to the participant’s main place of residence during each period. For questions referring to conditions ‘during the war,’ no specific year or phase of the conflict was predefined; participants were asked to report their overall experience during the conflict period. Medication access during the war was categorized as poor, defined as most medications being unavailable; moderate, defined as medications being sometimes available and sometimes unavailable; or easy, defined as medications being consistently available.

### Statistical analysis

Analyses were conducted using IBM SPSS Statistics for Windows, Version 26.0 (IBM Corp., Armonk, NY, USA). Descriptive statistics were used to summarize the characteristics of the study population and medication access-related variables. Categorical variables were presented as frequencies and percentages, while continuous variables were assessed using histograms, Q–Q plots, measures of skewness and kurtosis, and the Shapiro–Wilk test and summarized as mean with standard deviation or median with interquartile range, as appropriate. Because formal normality tests may detect minor deviations in large samples, greater emphasis was placed on graphical assessment and distributional shape when selecting summary statistics and analytical methods.

Chronic diseases were coded as multiple-response binary variables, with each chronic condition represented as a separate variable. A total chronic disease count and a binary indicator of the presence of any chronic disease were calculated for each participant. Likert-scale items assessing current medication access barriers and the impact of limited medication access were summarized using response frequencies and percentages. The seven-item medication access barrier score was treated as the primary analytical outcome. All seven barrier items were complete for all participants; therefore, no imputation was performed. Analyses were based on available data, and number of children was analyzed only among currently married participants for whom the variable was applicable.

Bivariate associations between participant characteristics and the medication access barrier score were examined using nonparametric methods. This approach was selected because the score was derived from ordinal Likert-scale items and did not fully satisfy parametric distributional assumptions. Spearman’s rank correlation was used to assess associations between the medication access barrier score and continuous predictors. The Mann–Whitney U test was used for binary categorical predictors, while the Kruskal–Wallis test was used for nominal categorical predictors with more than two groups. Ordered categorical predictors were analyzed using the Jonckheere–Terpstra trend test to assess monotonic trends across progressively higher or more favorable categories.

Effect sizes were reported as Spearman’s rho for correlation analyses, r for Mann–Whitney U tests calculated as |Z|/√N, and epsilon-squared for Kruskal–Wallis tests. For Jonckheere–Terpstra tests, effect size was calculated as *r* = Z/√N, with the sign retained to indicate the direction of the ordered trend. For chronic-disease variables with fewer than 10 participants in the ‘Yes’ category, two-sided Monte Carlo *p*-values based on 100,000 sampled tables were used. No pairwise post-hoc comparisons were performed for Kruskal–Wallis tests because none of the omnibus tests was statistically significant. Bivariate analyses were considered exploratory, and *p*-values were not adjusted for multiplicity across predictors.

A multivariable linear regression model was fitted to identify factors independently associated with the medication access barrier score. Candidate predictors were selected a priori according to conceptual relevance and data availability rather than solely on the basis of bivariate statistical significance. Age was modeled per 10-year increase. Distance to the nearest medication provider was log_2_-transformed because of its markedly right-skewed distribution; its coefficient therefore represented the expected change in the barrier score associated with each doubling of distance. The average and above-average economic status categories during the war were combined because the above-average subgroup contained only six participants. Number of children was excluded because it was applicable only to currently married participants, and rare individual chronic diseases were excluded because their small subgroup sizes could produce unstable adjusted estimates.

Model performance was summarized using the F statistic, R^2^, adjusted R^2^, and Cohen’s f^2^. Multicollinearity was evaluated using tolerance values and variance inflation factors. Residual histograms, normal probability plots, standardized residuals, leverage values, Mahalanobis distance, and Cook’s distance were examined to assess model assumptions and influential observations. The Ramsey RESET test was used to assess functional-form specification, and heteroscedasticity was evaluated using the Breusch–Pagan test. Because heteroscedasticity was detected, final statistical inference was based on robust sandwich standard errors and 95% Wald confidence intervals from a normal identity-link generalized linear model. Adjusted unstandardized coefficients, robust standard errors, 95% confidence intervals, and *p*-values were reported. Standardized beta coefficients and variance inflation factors were obtained from the corresponding ordinary least-squares model. A two-sided *p*-value < 0.05 was considered statistically significant.

## Results

### Participant flow and data completeness

A total of 1,054 responses were received. Twelve submissions (1.1%) were excluded during data cleaning because they contained substantial missing data or clearly contradictory response patterns across related questionnaire items that precluded reliable analysis. No duplicate or otherwise ineligible responses were identified. The final analytical sample therefore comprised 1,042 participants, representing 98.9% of the submitted responses. All variables included in the main analyses were complete, except for number of children, which was applicable only to the 447 currently married participants. No missing-value imputation was performed.

### Sociodemographic and socioeconomic characteristics

A total of 1,042 participants were included in the study. The median age was 26 years (IQR, 22–42), and females represented 62.0% of the sample. Most participants had university-level education or higher (73.1%), while 42.9% were currently married. Among currently married participants, the median number of children was 3 (IQR, 2–4). Before the war, 80.9% of the participants lived in Homs, compared with 74.7% during the war and 88.6% after the war. During the war, 50.0% lived in a family house, 28.3% in a rented house, and 21.7% in a private house. Employment status shifted from 29.8% employed before the war to 40.9% currently employed, while the proportion of students decreased from 50.8% to 35.2% (Table 1).

**Table 1.** Sociodemographic and socioeconomic characteristics of participants (N = 1,042).

Variable	Category / Statistic	Value
Age, years	Median (IQR)	26 (22–42)
Sex	Female	646 (62.0%)
	Male	396 (38.0%)
Educational level	Less than high school	129 (12.4%)
	High school diploma / Baccalaureate	151 (14.5%)
	University or higher	762 (73.1%)
Marital status	Currently unmarried	595 (57.1%)
	Currently married	447 (42.9%)
Number of children	Median (IQR), among valid responses	3 (2–4)
Residence before the war	In Homs	843 (80.9%)
	Outside Homs	199 (19.1%)
Residence during the war	In Homs	778 (74.7%)
	Outside Homs	264 (25.3%)
Residence after the war	In Homs	923 (88.6%)
	Outside Homs	119 (11.4%)
Housing status during the war	Family house	521 (50.0%)
	Rented house	295 (28.3%)
	Private house	226 (21.7%)
Economic status before the war	Poor	113 (10.8%)
	Below average	418 (40.1%)
	Average	361 (34.6%)
	Above average	150 (14.4%)
Economic status during the war	Poor	316 (30.3%)
	Below average	590 (56.6%)
	Average	130 (12.5%)
	Above average	6 (0.6%)
Employment status before the war	Employed	311 (29.8%)
	Student	529 (50.8%)
	Not currently employed	202 (19.4%)
Current employment status	Employed	426 (40.9%)
	Student	367 (35.2%)
	Not currently employed	249 (23.9%)

Note. Values are presented as n (%) unless otherwise indicated. Age and number of children are presented as median (interquartile range). Number of children was recorded only for currently married participants (valid n = 447) and was not applicable to currently unmarried participants (n = 595). All other variables had complete data (N = 1,042). Employment categories were grouped as follows: employed = full-time employed, part-time employed, or self-employed; not currently employed = homemaker, retired, or unemployed.

**Figure 1.**
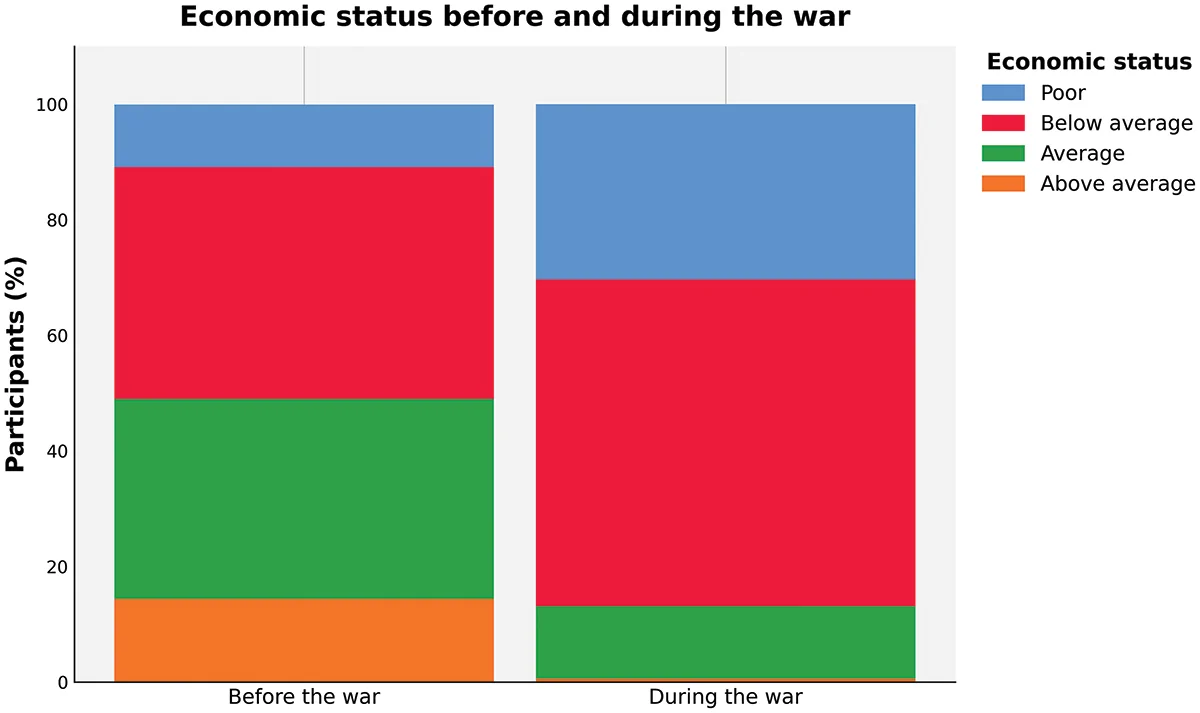
Change in economic status before and during the war. A 100% stacked bar chart showing the distribution of self-reported economic status before and during the war. The figure demonstrates an increase in poor and below-average economic status during the war, with a corresponding reduction in average and above-average economic status.

Self-reported economic status showed a marked deterioration during the war. The proportion of participants self-reporting poor economic status increased from 10.8% before the war to 30.3% during the war, while self-reported below-average economic status increased from 40.1% to 56.6%. In contrast, self-reported average economic status decreased from 34.6% to 12.5%, and self-reported above-average economic status decreased from 14.4% to 0.6%. These changes are illustrated in the 100% stacked bar chart (Figure 1).

### Chronic diseases and medication access during the war

Overall, 239 participants (22.9%) reported having at least one chronic disease. The most commonly reported chronic conditions were hypertension (9.0%), asthma (3.2%), chronic heart disease (3.0%), type 2 diabetes (2.9%), autoimmune disease (2.8%), and type 1 diabetes (2.7%). The median number of chronic diseases was 0 (IQR, 0–0), with an observed range of 0–9. Regarding access to medications during the war, 22.1% reported poor access, 67.0% reported moderate access, and only 10.9% reported easy access. The median distance to the nearest medication provider was 100 meters (IQR, 50–400) (Table 2).

**Table 2.** Chronic diseases and medication access during the war (N = 1,042).

Variable	Category / Statistic	Value
Any chronic disease	No	803 (77.1%)
	Yes	239 (22.9%)
Individual chronic diseases (multiple responses)	Asthma	33 (3.2%)
	Type 1 diabetes	28 (2.7%)
	Type 2 diabetes	30 (2.9%)
	Hypertension	94 (9.0%)
	Autoimmune disease	29 (2.8%)
	Chronic heart disease	31 (3.0%)
	Chronic liver disease	4 (0.4%)
	Chronic kidney disease	17 (1.6%)
	Chronic endocrine disease	22 (2.1%)
	Migraine/headache	7 (0.7%)
	Musculoskeletal disease	14 (1.3%)
	Allergy	6 (0.6%)
	Gastrointestinal disease	6 (0.6%)
	Anemia/blood disease	3 (0.3%)
	Cancer	3 (0.3%)
	Mental illness	3 (0.3%)
	Other chronic disease	11 (1.1%)
Number of chronic diseases	Median (IQR); observed range	0 (0–0); 0–9
Access to medications during the war	Poor (most medications unavailable)	230 (22.1%)
	Moderate (sometimes available, sometimes unavailable)	698 (67.0%)
	Easy (always available)	114 (10.9%)
Distance to nearest medication provider, meters	Median (IQR)	100 (50–400)

Note. Values are presented as n (%) unless otherwise indicated. Individual chronic diseases are shown as the number and percentage of participants reporting each condition; multiple responses were permitted, and percentages therefore do not sum to 100%. Number of chronic diseases and distance to the nearest medication provider are presented as median (interquartile range). All variables had complete data (N = 1,042). IQR, interquartile range.

### Current barriers to medication access

The seven-item medication access barrier score had a mean of 22.38 (SD, 4.84) and a median of 22 (IQR, 20–26), with possible and observed ranges of 7–35. The scale demonstrated acceptable internal consistency in the main sample, with a Cronbach’s alpha of 0.769. All corrected item-total correlations exceeded 0.30, and deletion of any item reduced the overall alpha, supporting retention of all seven items. Floor and ceiling values occurred in 0.8% and 0.5% of participants, respectively (Supplementary Table S1).

**Figure 2.**
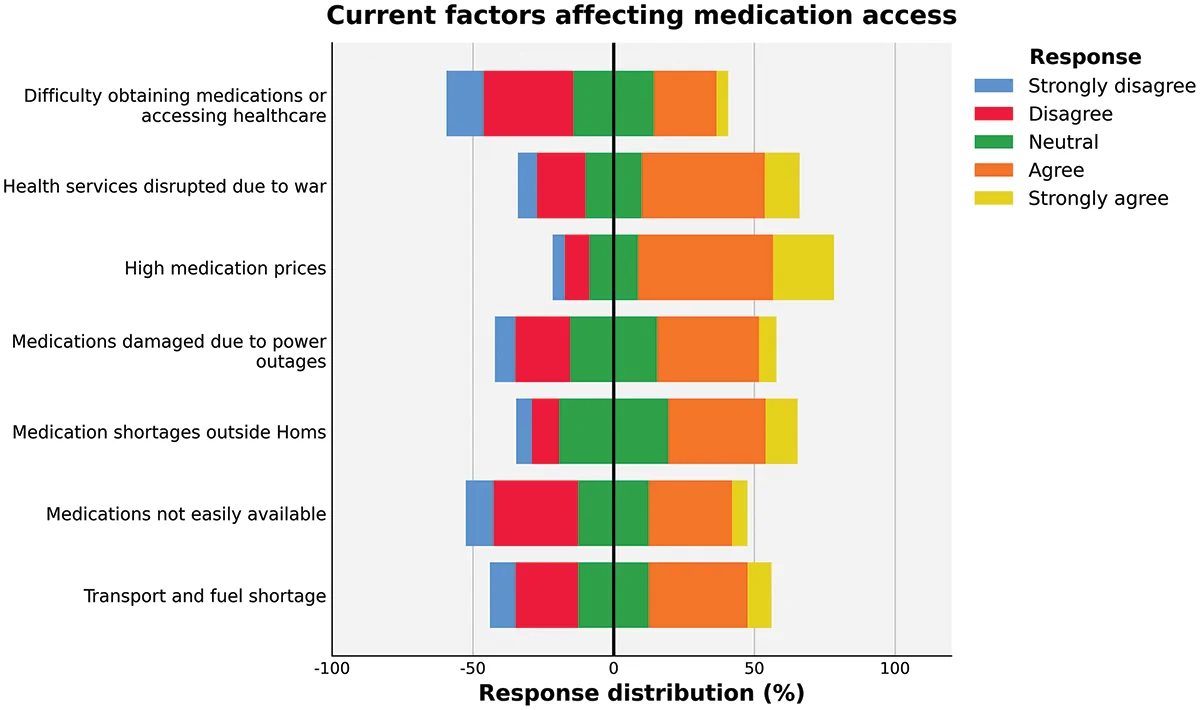
Current factors affecting medication access. Diverging stacked Likert chart showing participants’ responses to current medication access barriers. Negative values represent disagreement responses displayed to the left of the neutral midpoint for visualization only; all values represent percentages.

The most frequently endorsed barrier was the high cost of medications, with 69.7% of participants agreeing or strongly agreeing that medication prices made access difficult. More than half of participants agreed or strongly agreed that health services in their area had been disrupted or stopped due to the war (56.1%). Other commonly reported barriers included medication shortages outside Homs (46.1%), transportation and fuel shortages limiting access to healthcare facilities (**43.7%**), and medication damage due to power outages (42.4%) (Table 3 and Figure 2).

**Table 3.** Current factors affecting medication access among participants (N = 1,042).

Variable	Strongly disagree n (%)	Disagree n (%)	Neutral n (%)	Agree n (%)	Strongly agree n (%)
Difficulty obtaining medications or accessing healthcare facilities	136 (13.1%)	333 (32.0%)	297 (28.5%)	232 (22.3%)	44 (4.2%)
Health services in the area were disrupted or stopped due to the war	71 (6.8%)	180 (17.3%)	206 (19.8%)	456 (43.8%)	129 (12.4%)
Medications in the area were damaged due to power outages	75 (7.2%)	203 (19.5%)	322 (30.9%)	377 (36.2%)	65 (6.2%)
Medications were not easily available in the area	102 (9.8%)	315 (30.2%)	261 (25.0%)	308 (29.6%)	56 (5.4%)
Difficulty obtaining medications due to high prices	45 (4.3%)	91 (8.7%)	180 (17.3%)	500 (48.0%)	226 (21.7%)
Difficulty accessing healthcare facilities due to shortages of transportation and fuel	94 (9.0%)	233 (22.4%)	260 (25.0%)	366 (35.1%)	89 (8.5%)
Pharmacies outside Homs have medication shortages	58 (5.6%)	100 (9.6%)	404 (38.8%)	361 (34.6%)	119 (11.4%)
Medication access barrier score			Median (IQR): 22 (20–26)		

Note. Responses were coded from 1 (strongly disagree) to 5 (strongly agree). The medication access barrier score was calculated by summing all seven items; higher scores indicate greater perceived barriers. All seven items were complete for all participants, so no imputation was required. The possible and observed ranges were both 7–35. Mean (SD) was 22.38 (4.84), median (IQR) was 22 (20–26), and Cronbach’s alpha was 0.769. Floor and ceiling values occurred in 0.8% and 0.5% of participants, respectively. IQR, interquartile range; SD, standard deviation.

### Perceived consequences of limited medication access

**Figure 3.**
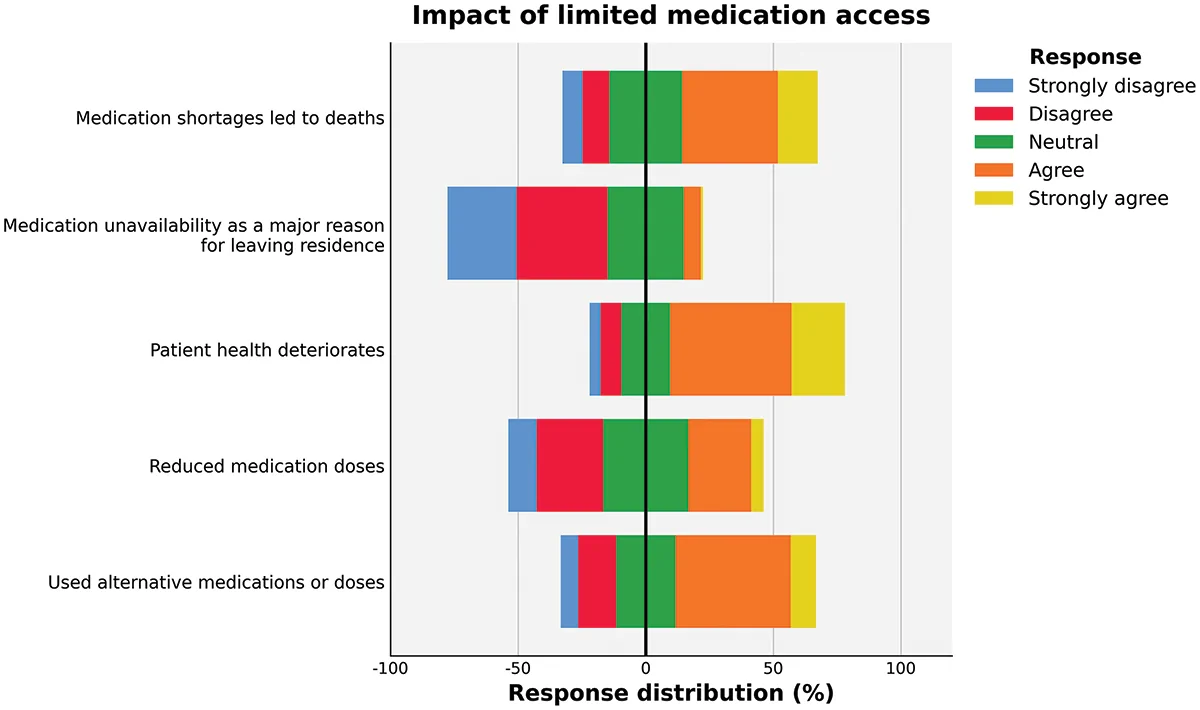
Perceived impact of limited medication access. Diverging stacked Likert chart showing participants’ perceptions of the consequences of limited medication access. Negative values represent disagreement responses displayed to the left of the neutral midpoint for visualization only; all values represent percentages.

Most participants agreed or strongly agreed with the statement that patients’ health deteriorated because of an inability to access medications (68.5%). More than half agreed or strongly agreed that medication unavailability led to the use of alternative medications or alternative doses (55.2%), while 53.2% agreed or strongly agreed that medication shortages had led to deaths. Reduced medication doses because of shortages were reported by 29.5%. Medication unavailability was less commonly identified as a major reason for leaving the place of residence, with 7.5% agreeing or strongly agreeing with this statement (Table 4 and Figure 3).

**Table 4.** Impact of limited medication access among participants (N = 1,042).

Variable	Strongly disagree n (%)	Disagree n (%)	Neutral n (%)	Agree n (%)	Strongly agree n (%)
Medication unavailability was a major reason for leaving the place of residence	281 (27.0%)	372 (35.7%)	311 (29.8%)	70 (6.7%)	8 (0.8%)
Use of alternative medications or alternative doses due to medication unavailability	71 (6.8%)	155 (14.9%)	241 (23.1%)	472 (45.3%)	103 (9.9%)
Reduced medication doses due to medication shortages	116 (11.1%)	271 (26.0%)	348 (33.4%)	256 (24.6%)	51 (4.9%)
Patient health deteriorates due to inability to access medications	45 (4.3%)	84 (8.1%)	199 (19.1%)	496 (47.6%)	218 (20.9%)
Medication shortages led to deaths	82 (7.9%)	108 (10.4%)	298 (28.6%)	391 (37.5%)	163 (15.6%)

Note. Values are presented as n (%). All five items were complete for all 1,042 participants. Responses were coded from 1 (strongly disagree) to 5 (strongly agree) and were analysed individually; no composite consequence score was calculated or used in subsequent analyses. Percentages may not sum to exactly 100% because of rounding.

Exact combined agreement (agree + strongly agree): displacement, 78 (7.5%); alternative medications or doses, 575 (55.2%); reduced doses, 307 (29.5%); health deterioration, 714 (68.5%); deaths, 554 (53.2%).

### Bivariate associations with the medication access barrier score

Among continuous predictors, distance to the nearest medication provider was positively associated with the medication access barrier score (Spearman’s ρ = 0.199, *p* < 0.001). Age, number of children, and number of chronic diseases were not significantly associated with the score. Participants who had lived outside Homs before the war had higher mean ranks than those who had lived in Homs (569.38 vs 510.20; U = 74,349.5; Z = −2.501; *r* = 0.077; *p* = 0.012). Residence during and after the war, sex, marital status, and the presence of any chronic disease were not significantly associated with the barrier score.

Ordered trend analyses showed that progressively more favorable economic status before the war was associated with lower barrier scores (Jonckheere–Terpstra J = 173,883.0; Z = −2.365; *r* = −0.073; *p* = 0.018). A stronger trend was observed for economic status during the war (J = 130,355.0; Z = −4.926; *r* = −0.153; *p* < 0.001), with the highest mean rank among participants reporting poor economic status and the lowest among those reporting average or above-average status. Access to medications during the war showed the strongest ordered association: mean ranks decreased from 643.81 among participants reporting poor access to 511.57 among those reporting moderate access and 335.55 among those reporting easy access (J = 90,859.0; Z = −9.120; *r* = −0.283; *p* < 0.001). Educational level did not show a significant ordered trend, and none of the Kruskal–Wallis tests for housing or employment status was statistically significant.

Exploratory analyses produced nominally significant findings for chronic liver disease (*n* = 4; *p* = 0.037) and gastrointestinal disease (*n* = 6; *p* = 0.015). Because these findings were based on extremely small subgroups and the analyses were not adjusted for multiplicity, they should be interpreted with considerable caution. Other individual chronic diseases were not significantly associated with the barrier score. Complete bivariate findings, including nonsignificant associations, are reported in Table 5.

**Table 5.** Bivariate associations between participant characteristics and the seven-item medication access barrier score (N = 1,042).

Variable	Category / measure	n	Mean rank / rho	Test statistic	Effect size	p-value
A. Continuous predictors – Spearman rank correlation						
Age	Continuous	1042	rho = −0.057	Spearman rho = −0.057	rho = −0.057	0.068
Number of children	Continuous	447	rho = −0.030	Spearman rho = −0.030	rho = −0.030	0.530
Number of chronic diseases	Continuous	1042	rho = 0.012	Spearman rho = 0.012	rho = 0.012	0.687
Distance to nearest medication provider, m	Continuous	1042	rho = 0.199	Spearman rho = 0.199	rho = 0.199	<0.001
B. Binary predictors – Mann–Whitney U tests						
Sex	Female	646	518.45	U = 125,938.5; Z = −0.419	r = 0.013	0.675
	Male	396	526.47			
Marital status	Currently unmarried	595	522.10	U = 132,625.0; Z = −0.075	r = 0.002	0.941
	Currently married	447	520.70			
Residence before the war	Outside Homs	199	569.38	U = 74,349.5; Z = −2.501	r = 0.077	0.012
	In Homs	843	510.20			
Residence during the war	Outside Homs	264	548.28	U = 95,626.0; Z = −1.677	r = 0.052	0.093
	In Homs	778	512.41			
Residence after the war	Outside Homs	119	565.00	U = 49,741.5; Z = −1.680	r = 0.052	0.093
	In Homs	923	515.89			
Any chronic disease	No	803	518.52	U = 93,566.5; Z = −0.587	r = 0.018	0.557
	Yes	239	531.51			
Asthma	No	1009	523.10	U = 15,035.0; Z = −0.951	r = 0.029	0.342
	Yes	33	472.61			
Type 1 diabetes	No	1014	521.68	U = 14,014.0; Z = −0.116	r = 0.004	0.908
	Yes	28	515.00			
Type 2 diabetes	No	1012	524.54	U = 12,102.0; Z = −1.899	r = 0.059	0.058
	Yes	30	418.90			
Hypertension	No	948	519.60	U = 42,753.0; Z = −0.649	r = 0.020	0.516
	Yes	94	540.68			
Autoimmune disease	No	1013	522.37	U = 13,806.0; Z = −0.554	r = 0.017	0.580
	Yes	29	491.07			
Chronic heart disease	No	1011	521.31	U = 15,476.0; Z = −0.118	r = 0.004	0.906
	Yes	31	527.77			
Chronic liver disease	No	1038	522.68	U = 847.5; Z = −2.050	r = 0.064	0.037 (MC)
	Yes	4	214.38			
Chronic kidney disease	No	1025	521.22	U = 8,422.0; Z = −0.237	r = 0.007	0.813
	Yes	17	538.59			
Chronic endocrine disease	No	1020	521.85	U = 10,866.5; Z = −0.254	r = 0.008	0.800
	Yes	22	505.43			
Migraine / headache	No	1035	521.56	U = 3,560.5; Z = −0.078	r = 0.002	0.939 (MC)
	Yes	7	512.64			
Musculoskeletal disease	No	1028	521.64	U = 7,055.5; Z = −0.126	r = 0.004	0.900
	Yes	14	511.46			
Allergy	No	1036	522.42	U = 2,159.5; Z = −1.293	r = 0.040	0.201 (MC)
	Yes	6	363.42			
Gastrointestinal disease	No	1036	523.18	U = 1,363.5; Z = −2.379	r = 0.074	0.015 (MC)
	Yes	6	230.75			
Anemia / blood disease	No	1039	520.79	U = 818.5; Z = −1.425	r = 0.044	0.161 (MC)
	Yes	3	768.17			
Cancer	No	1039	521.18	U = 1,228.0; Z = −0.636	r = 0.020	0.548 (MC)
	Yes	3	631.67			
Mental illness	No	1039	521.88	U = 1,167.0; Z = −0.754	r = 0.023	0.474 (MC)
	Yes	3	391.00			
Other chronic disease	No	1031	522.18	U = 4,968.5; Z = −0.709	r = 0.022	0.478
	Yes	11	457.68			
C. Nominal predictors with more than two categories – Kruskal-Wallis tests						
Housing status during the war	Family house	521	514.53	H = 4.191; df = 2	epsilon-squared = 0.002	0.123
	Rented house	295	550.41			
	Private house	226	499.84			
Employment status before the war	Employed	311	515.93	H = 0.694; df = 2	epsilon-squared = 0.000	0.707
	Student	529	528.92			
	Not currently employed	202	510.65			
Current employment status	Employed	426	498.91	H = 5.015; df = 2	epsilon-squared = 0.003	0.081
	Student	367	546.76			
	Not currently employed	249	522.92			
D. Ordered categorical predictors – Jonckheere-Terpstra trend tests						
Educational level	Less than high school	129	512.14	J = 118,552.0; Z = 0.490	r = 0.015	0.624
	High school / baccalaureate	151	515.77			
	University or higher	762	524.22			
Economic status before the war	Poor	113	570.40	J = 173,883.0; Z = −2.365	r = −0.073	0.018
	Below average	418	535.23			
	Average	361	496.52			
	Above average	150	506.50			
Economic status during the war	Poor	316	587.75	J = 130,355.0; Z = −4.926	r = −0.153	<0.001
	Below average	590	500.65			
	Average or above	136	458.04			
Access to medications during the war	Poor	230	643.81	J = 90,859.0; Z = −9.120	r = −0.283	<0.001
	Moderate	698	511.57			
	Easy	114	335.55			

Outcome: seven-item medication access barrier score (possible range, 7–35); higher scores indicate greater perceived barriers.

Note. Higher mean ranks indicate higher medication-access barrier scores. Continuous predictors were assessed using Spearman rank correlation. Binary predictors were assessed using Mann-Whitney U tests; effect size was calculated as r = |Z|/sqrt(N). Nominal predictors with more than two categories were assessed using Kruskal-Wallis tests; epsilon-squared was calculated as (H – k + 1)/(N – k), with negative values set to 0. Ordered categorical predictors were assessed using the Jonckheere-Terpstra trend test; effect size was calculated as r = Z/sqrt(N). Negative Jonckheere-Terpstra effect sizes indicate decreasing barrier scores across progressively higher or more favorable categories. All reported p-values are two-sided. MC indicates a two-sided Monte Carlo p-value based on 100,000 sampled tables for chronic-disease groups with fewer than 10 participants in the Yes category. Number of children was available only for currently married participants (valid n = 447). Average and above-average wartime economic status were combined because the above-average subgroup was small (n = 6). No pairwise post-hoc comparisons are reported: all Kruskal-Wallis omnibus tests were nonsignificant, and ordered analyses focused on the overall trend. Bivariate analyses were exploratory and p-values were not adjusted for multiplicity across predictors. Findings involving very rare chronic diseases should be interpreted cautiously.

## Multivariable analysis

The multivariable ordinary least-squares model was statistically significant, F(19, 1022) = 4.228, *p* < 0.001, and explained 7.3% of the variance in the medication access barrier score (R^2^ = 0.073; adjusted R^2^ = 0.056; Cohen’s f^2^ = 0.079). The corresponding robust-covariance model also remained statistically significant (likelihood-ratio χ^2^(19) = 78.841, *p* < 0.001). Because the Breusch–Pagan test indicated heteroscedasticity (BP = 38.55, df = 19, *p* = 0.005), inference was based on robust sandwich standard errors and 95% Wald confidence intervals.

Four variables were independently associated with the barrier score. Participants reporting poor economic status during the war had scores that were, on average, 1.900 points higher than those reporting average or above-average status (adjusted B = 1.900; robust SE = 0.538; 95% CI, 0.845 to 2.955; *p* < 0.001). Students had scores 0.861 points higher than employed participants (adjusted B = 0.861; robust SE = 0.389; 95% CI, 0.099 to 1.623; *p* = 0.027). Each doubling of the distance to the nearest medication provider was associated with a 0.345-point increase in the barrier score (adjusted B = 0.345; robust SE = 0.063; 95% CI, 0.221 to 0.469; *p* < 0.001). Participants with less than high-school education had scores 1.258 points lower than those with university-level education or higher (adjusted B = −1.258; robust SE = 0.612; 95% CI, −2.457 to −0.059; *p* = 0.040). No other demographic, health, residence, housing, economic, or employment variable was independently associated with the outcome (Table 6).

**Table 6.** Multivariable linear regression of factors associated with the seven-item medication access barrier score (N = 1,042).

Predictor	Adjusted B	Robust SE	95% Wald CI	Std. beta	p-value
Intercept	20.919	0.546	19.849 to 21.989	–	**<0.001**
Age, per 10-year increase	−0.293	0.195	−0.674 to 0.089	−0.085	0.132
Male (reference: female)	−0.061	0.310	−0.669 to 0.547	−0.006	0.844
Currently married (reference: currently unmarried)	0.706	0.469	−0.213 to 1.625	0.072	0.132
Less than high school (reference: university or higher)	−1.258	0.612	−2.457 to −0.059	−0.086	**0.040**
High school / baccalaureate (reference: university or higher)	−0.584	0.454	−1.474 to 0.305	−0.043	0.198
Any chronic disease (reference: none)	0.182	0.436	−0.673 to 1.037	0.016	0.677
Outside Homs before the war (reference: in Homs)	0.736	0.502	−0.248 to 1.721	0.060	0.143
Outside Homs during the war (reference: in Homs)	0.156	0.459	−0.743 to 1.056	0.014	0.733
Outside Homs after the war (reference: in Homs)	−0.101	0.571	−1.219 to 1.017	−0.007	0.860
Rented house during the war (reference: family house)	0.068	0.368	−0.653 to 0.788	0.006	0.854
Private house during the war (reference: family house)	−0.255	0.415	−1.068 to 0.557	−0.022	0.538
Poor economic status before the war (reference: average)	0.099	0.658	−1.191 to 1.390	0.006	0.880
Below-average economic status before the war (reference: average)	0.117	0.351	−0.571 to 0.805	0.012	0.740
Above-average economic status before the war (reference: average)	0.150	0.451	−0.734 to 1.034	0.011	0.740
Poor economic status during the war (reference: average or above)	1.900	0.538	0.845 to 2.955	0.180	**<0.001**
Below-average economic status during the war (reference: average or above)	0.519	0.446	−0.356 to 1.394	0.053	0.245
Student (reference: employed)	0.861	0.389	0.099 to 1.623	0.085	**0.027**
Not currently employed (reference: employed)	0.405	0.434	−0.445 to 1.255	0.036	0.351
Distance to nearest medication provider, per doubling	0.345	0.063	0.221 to 0.469	0.177	**<0.001**

Adjusted unstandardized coefficients are shown with robust sandwich standard errors and 95% Wald confidence intervals.

The multivariable OLS model was statistically significant, F(19, 1022) = 4.228, *p* < 0.001, and explained 7.3% of the variance in the seven-item medication access barrier score (R^2^ = 0.073; adjusted R^2^ = 0.056; Cohen’s f^2^ = 0.079), indicating a small explanatory effect. The robust-covariance model also remained statistically significant, likelihood-ratio χ^2^(19) = 78.841, *p* < 0.001. The Breusch–Pagan test indicated heteroscedasticity (BP = 38.55, df = 19, *p* = 0.005); therefore, robust sandwich standard errors and 95% Wald confidence intervals were used for inference. The RESET test did not indicate important functional-form misspecification (ΔR^2^ = 0.003; F-change(2, 1020) = 1.484; *p* = 0.227). No concerning multicollinearity was detected, with a maximum variance inflation factor of 3.038, and no observation exerted excessive influence, with a maximum Cook’s distance of 0.0235 and no value exceeding 1. The dependent variable was the seven-item medication access barrier score, ranging from 7 to 35, with higher scores indicating greater barriers. Adjusted B values are unstandardized coefficients. Standardized beta coefficients and variance inflation factors were obtained from the corresponding OLS model. Age was modeled per 10-year increase, while distance was log_2_-transformed, so its coefficient represents the change in barrier score associated with each doubling of distance. Economic status during the war was grouped as poor, below average, and average or above because the original above-average category included only six participants. Number of children was excluded because it was applicable only to currently married participants, and rare individual chronic diseases were not entered because their small group sizes would have produced unstable multivariable estimates.

Model diagnostics did not indicate concerning multicollinearity, with a maximum variance inflation factor of 3.038. The RESET test did not suggest important functional-form misspecification (ΔR^2^ = 0.003; F-change(2, 1020) = 1.484; *p* = 0.227). Seven participants (0.7%) had absolute standardized residuals of at least 3, but no observation exerted excessive influence; the maximum Cook’s distance was 0.0235, and no value exceeded 1.

## Discussion

This study provides community-level evidence on perceived medication access barriers among residents of Homs during and after the war. Only 10.9% of the participants described medication access during the war as easy, and high medication prices were the most frequently endorsed current barrier. Disruption of health services, medication shortages outside Homs, transportation and fuel shortages, and medication damage related to power outages were also commonly reported. In the adjusted analysis, self-reported poor economic status during the war, student status, and greater distance to the nearest medication provider were associated with higher medication access barrier scores, whereas less than high-school education was associated with lower scores. Participants also perceived limited medication access as contributing to health deterioration, alternative medication or dose use, reduced dosing, and deaths.

These findings should be interpreted within the broader deterioration of Syria’s health system and socioeconomic conditions. WHO’s 2025 Health Emergency Appeal reported that almost 15 million people in Syria needed urgent health care assistance in 2024, with this number expected to rise to 16.7 million in 2025 [15]. OCHA also reported that 90% of Syrians were living below the poverty line, forcing many households to reduce spending on essentials such as food, water, healthcare, and education [16]. In the health sector, disruption in the supply of medicines and medical equipment remains critical, particularly for essential medicines used for chronic conditions, including noncommunicable diseases [16]. The present findings add local evidence from Homs regarding how residents experienced these wider economic and health-system pressures.

The findings concerning self-reported economic status provide an important context for interpreting medication access barriers. In our sample, the proportion reporting poor economic status increased from 10.8% before the war to 30.3% during the war, while the proportion reporting below-average status increased from 40.1% to 56.6%. At the same time, the proportions reporting average and above-average economic status declined markedly. This pattern supports the interpretation that medication access was affected not only by the physical availability of medicines but also by household purchasing power. This interpretation was further supported by the multivariable analysis, in which participants reporting poor economic status during the war had barrier scores that were, on average, 1.90 points higher than those reporting average or above-average status. This is consistent with previous literature on conflict-affected Syrian populations, where cost repeatedly appears as a major barrier to healthcare and chronic disease treatment. A review of noncommunicable disease care among Syrian refugees in Jordan, Lebanon, and Turkey reported that treatment cost was the primary obstacle to NCD care, with medication costs contributing substantially to out-of-pocket spending and treatment interruption [17]. Similarly, studies among Syrian refugees in Jordan and Lebanon found that cost influenced care-seeking, facility choice, medication access, and adherence [18,19].

The central role of affordability in our study is further supported by the finding that high medication prices were endorsed by 69.7% of the participants, making it the most frequently reported current barrier. More than half also reported disruption or stoppage of health services in their area due to the war, while transportation and fuel shortages, medication shortages outside Homs, and medication damage due to power outages were also common. These findings align with recent conflict-setting studies showing that war can reduce both the affordability and availability of essential medicines. In Northern Syria, Aljadeeah et al. reported low availability of essential medicines for noncommunicable diseases, with shortages and price variation linked to geopolitical tension, disrupted supply chains, weak regulation, infrastructure destruction, trade restrictions, and economic instability [7]. In Sudan, Ahmed et al. found that essential medicine availability in primary healthcare centers was only 45.1%, and that war affected participants’ ability to obtain medications, reduced medication availability, increased transportation difficulties, and increased medication prices [20]. Together, these comparisons suggest that the barriers reported in Homs reflect interacting problems of affordability, medicine availability, service disruption, transportation, and infrastructure rather than an isolated shortage at the pharmacy level.

This interpretation is also consistent with reviews of the Syrian health system, which describe workforce shortages, uneven service distribution, financing difficulties, supply-chain disruption, and increased reliance on private-sector provision [21,22]. A qualitative study of attacks on healthcare in Syria similarly described cascading effects on service delivery, health governance, workforce, financing, infrastructure, and community health [8]. However, these dimensions were not formally derived or tested as separate categories in the present study and should be understood as an interpretive framework informed by the reported items and existing literature.

Geographic access also remained relevant after adjustment. Each doubling of the self-estimated distance to the nearest medication provider was associated with a 0.345-point increase in the medication access barrier score. Although residence outside Homs before the war was associated with higher scores in the bivariate analysis, residence before, during, or after the war was not independently associated with the outcome in the multivariable model. A systematic review of pharmacy mapping studies emphasized that access to medicines is often measured through availability and affordability, while geographic accessibility remains an important but less frequently assessed dimension [23]. Transportation barriers are also known to affect healthcare access and medication use, especially among lower-income and vulnerable populations, and may lead to delayed care, missed appointments, and missed or delayed medication use [24]. In our setting, transportation and fuel shortages likely interacted with economic hardship, making even physically available medicines difficult to obtain for some participants.

The perceived consequences of limited medication access were substantial. Most participants agreed with the statement that patients’ health deteriorated because of an inability to access medications, while more than half agreed that medication unavailability resulted in alternative medication or dose use and that medication shortages had led to deaths. These responses represent participants’ perceptions and were not verified using mortality records, clinical records, pharmacy data, or verbal autopsy. These findings are comparable to the Sudanese medication-access study, where 65.7% of the participants reported health deterioration due to lack of medication, 42.7% used alternative drugs or adjusted doses, 33.3% reduced medication intake, and 61.3% believed medication shortages contributed to deaths [6].

The multivariable analysis identified two additional findings requiring cautious interpretation. Students had slightly higher barrier scores than employed participants, although the adjusted difference was small. Participants with less than high-school education had lower scores than those with university-level education or higher, despite the absence of a significant ordered educational trend in the bivariate analysis. This unexpected association may reflect differences in the recognition or reporting of barriers, the highly educated composition of the sample, residual confounding, or sampling variability and should not be interpreted as evidence that lower education protects against medication access problems.

The adjusted model explained 5.6% of the variance in barrier scores. This modest explanatory capacity indicates that the measured sociodemographic, economic, residence, and geographic variables captured only a limited proportion of the variation in perceived barriers. Other unmeasured factors, such as household income, medication type, disease severity, local pharmacy availability, insurance coverage, informal support networks, and changes in medicine prices over time, may also be important. Accordingly, statistically significant coefficients should be interpreted as modest associations rather than strong independent determinants.

These findings are particularly relevant to the current efforts to restore and reorganize Syria’s health system. The Syrian National Health Compact emphasizes infrastructure rehabilitation, service readiness, health financing, supply-chain resilience, and reduced financial hardship as part of the country’s recovery trajectory [25]. Although the present study did not directly evaluate specific policies or interventions, the findings, considered alongside existing literature, suggest several potential priorities for policymakers. Recovery planning focused only on rebuilding facilities or restocking medicines may fail to resolve the barriers experienced by patients if medicines remain unaffordable, transportation remains difficult, electricity-dependent storage remains unreliable, or peripheral areas remain underserved. In Homs and similar post-conflict settings, medication-access strategies **could** combine supply-side and demand-side interventions. These may include reliable procurement pathways, strengthened distribution to peripheral pharmacies and health facilities, protection of electricity-dependent medication storage, transportation-sensitive service planning, and financial protection measures such as price regulation, targeted subsidies, or insurance-based support for essential chronic medications. Rebuilding and strengthening primary healthcare services may also support treatment continuity by bringing essential medicines and routine follow-up closer to affected communities [25]. These recommendations are potential implications informed by the study findings and existing evidence and were not directly tested in the present analysis.

This study has several limitations. Its cross-sectional design prevents causal inference, and several variables relied on retrospective self-report about conditions before and during the war, which may introduce recall bias. Because no specific year or phase of the conflict was predefined for questions referring to conditions ‘during the war,’ participants may have used different temporal reference points across the prolonged conflict, introducing additional temporal-classification uncertainty. Economic status was based on subjective self-classification rather than objective income measures and may therefore be affected by reporting differences. Medication access barriers and consequences were self-reported, and perceived health deterioration, deaths, alternative dosing, and medication substitution were not verified through clinical records, pharmacy records, or facility-level medicine availability data. Distance to the nearest medication provider was also self-estimated and may have been measured imprecisely.

The use of convenience sampling through online networks, hospitals, and public places may have introduced selection bias. Accordingly, the 95% confidence level and 5% margin of error used for recruitment planning should not be interpreted as population-level precision for Homs. The high proportion of participants with university-level education or higher suggests that the sample may overrepresent younger, more educated, or more digitally connected residents and may not reflect the broader population of Homs. Although paper and interviewer-administered questionnaires were used to include individuals with limited internet access or difficulty reading, the mode of questionnaire completion was not recorded. We could therefore not compare respondents across collection modes or assess whether mode-related differences influenced responses. Interviewer administration may also have introduced social-desirability or interviewer-related information bias despite the use of standardized wording.

The findings are also specific to Homs and may not be generalizable to all regions of Syria, where health-system control, infrastructure damage, displacement patterns, and medicine supply chains may differ.

This study also has several strengths. It provides local evidence from Homs, a setting where medication-access data remain limited despite the long-term effects of conflict on health services. The sample was relatively large, and the questionnaire captured multiple dimensions of medication access, including affordability, geographic access, service disruption, transportation, medication storage conditions, and perceived consequences. The seven-item medication access barrier score demonstrated acceptable internal consistency, and the use of comprehensive bivariate analyses, multivariable modeling with robust standard errors, and formal model diagnostics strengthened the analytical approach. These features make the findings useful not only for describing medication barriers, but also for identifying practical access points for health-system recovery.

## Conclusions

Medication access problems were commonly reported among this sample of residents of Homs. High medication prices, disrupted health services, transportation and fuel shortages, medication shortages outside Homs, and power-related medication damage were among the most frequently perceived barriers. Self-reported poor economic status during the war, student status, and greater distance to the nearest medication provider were independently associated with higher medication access barrier scores, while the inverse association observed for lower educational attainment requires cautious interpretation.

Participants also perceived limited medication access as contributing to health deterioration, alternative medication or dose use, reduced dosing, and deaths. The findings may inform post-conflict planning aimed at improving medicine affordability, strengthening pharmaceutical procurement and distribution, restoring primary healthcare services, supporting reliable medication storage, and reducing geographic and transportation barriers.

## Supplementary Material

STROBE Checklist.pdf

Supplementary Table S1.docx

## Data Availability

The data that support the findings of this study are available from the corresponding author upon reasonable request.

## Preprints

This manuscript has not been posted as a preprint.
