# Supplementary material for: Medication access barriers and perceived health consequences in post-war Syria: a cross-sectional study in Homs
Source: Glob Health Action. 2026 Sep 4;19(1):2726661. doi: 10.1080/16549716.2026.2726661 (PMC13552326; doi:10.1080/16549716.2026.2726661)
Supplement: Supplementary file 1 [file ZGHA_A_2726661_SM7490.pdf]

STROBE Statement—Checklist of items for cross-sectional studies

Section	Item no.	STROBE recommendation	Reported in manuscript
Title and abstract	1(a)	Indicate the study's design with a commonly used term in the title or abstract.	Title and Abstract, pp. 1–4
Title and abstract	1(b)	Provide an informative and balanced summary of what was done and what was found.	Abstract, pp. 3–4
Introduction	2	Explain the scientific background and rationale for the investigation being reported.	Background, pp. 4–5
Introduction	3	State specific objectives, including any prespecified hypotheses.	Background, final paragraph, p. 5
Methods	4	Present key elements of study design early in the paper.	Methods: Study design, pp. 5–6
Methods	5	Describe the setting, locations, and relevant dates, including periods of recruitment, exposure, follow-up, and data collection.	Methods: Study design, pp. 5–6
Methods	6(a)	Give the eligibility criteria, and the sources and methods of selection of participants.	Methods: Study design and Inclusion and exclusion criteria, pp. 6–7
Methods	6(b)	For matched studies, give matching criteria and number of exposed and unexposed.	Not applicable (unmatched cross-sectional study)
Methods	7	Clearly define all outcomes, exposures, predictors, potential confounders, and effect modifiers. Give diagnostic criteria, if applicable.	Methods: Measurement tools and Statistical analysis, pp. 7–11
Methods	8	For each variable of interest, give sources of data and details of methods of assessment or measurement. Describe comparability of assessment methods if there is more than one group.	Methods: Measurement tools, pp. 7–9
Methods	9	Describe any efforts to address potential sources of bias.	Methods: Study design and Measurement tools, pp. 5–9; Discussion: Limitations, pp. 20–21
Methods	10	Explain how the study size was arrived at.	Methods: Study design, p. 6

Section	Item no.	STROBE recommendation	Reported in manuscript
Methods	11	Explain how quantitative variables were handled in the analyses. If applicable, describe which groupings were chosen and why.	Methods: Statistical analysis, pp. 9–11
Methods	12(a)	Describe all statistical methods, including those used to control for confounding.	Methods: Statistical analysis, pp. 9–11
Methods	12(b)	Describe any methods used to examine subgroups and interactions.	Not applicable (no prespecified interaction analyses)
Methods	12(c)	Explain how missing data were addressed.	Methods: Inclusion and exclusion criteria and Statistical analysis, pp. 7 and 9; Results: Participant flow and data completeness, p. 11
Methods	12(d)	If applicable, describe analytical methods taking account of sampling strategy.	Not applicable (convenience sample; no probability-based sampling design or weighting)
Methods	12(e)	Describe any sensitivity analyses.	Not applicable (no formal sensitivity analyses)
Results	13(a)	Report numbers of individuals at each stage of study, such as potentially eligible, examined for eligibility, confirmed eligible, included, completing follow-up, and analysed.	Results: Participant flow and data completeness, p. 11
Results	13(b)	Give reasons for non-participation at each stage.	Results: Participant flow and data completeness, p. 11 (reasons for exclusion); non-participation during convenience recruitment was not quantified
Results	13(c)	Consider use of a flow diagram.	Not applicable; participant flow is reported in text
Results	14(a)	Give characteristics of study participants and information on exposures and potential confounders.	Results, pp. 12–13; Tables 1–2
Results	14(b)	Indicate number of participants with missing data for each variable of interest.	Results: Participant flow and data completeness, p. 11; Tables 1–4
Results	15	Report numbers of outcome events or summary measures.	Results, pp. 13–14; Tables 3–4; Figures 2–3

Section	Item no.	STROBE recommendation	Reported in manuscript
Results	16(a)	Give unadjusted estimates and, if applicable, confounder-adjusted estimates and their precision. Make clear which confounders were adjusted for and why they were included.	Results: Bivariate associations and Multivariable analysis, pp. 14–15; Tables 5–6
Results	16(b)	Report category boundaries when continuous variables were categorized.	Not applicable; continuous variables were not categorized in the main adjusted model. Scaling and transformation are described in Methods, pp. 10–11
Results	16(c)	If relevant, consider translating estimates of relative risk into absolute risk for a meaningful time period.	Not applicable
Results	17	Report other analyses done, such as analyses of subgroups and interactions, and sensitivity analyses.	Results, pp. 14–15; Table 5; Table 6; Supplementary Table S1
Discussion	18	Summarise key results with reference to study objectives.	Discussion, opening paragraph, p. 15
Discussion	19	Discuss limitations of the study, taking into account sources of potential bias or imprecision. Discuss both direction and magnitude of any potential bias.	Discussion: Limitations, pp. 20–21
Discussion	20	Give a cautious overall interpretation of results considering objectives, limitations, multiplicity of analyses, results from similar studies, and other relevant evidence.	Discussion, pp. 15–21
Discussion	21	Discuss the generalisability or external validity of the study results.	Discussion: Limitations, pp. 20–21
Other information	22	Give the source of funding and the role of the funders for the present study and, if applicable, for the original study on which the present article is based.	Funding information, p. 23
