# Supplementary material for: Medication access barriers and perceived health consequences in post-war Syria: a cross-sectional study in Homs
Source: Glob Health Action. 2026 Sep 4;19(1):2726661. doi: 10.1080/16549716.2026.2726661 (PMC13552326; doi:10.1080/16549716.2026.2726661)
Supplement: Supplementary file 2 [file ZGHA_A_2726661_SM7489.docx]

**Supplementary Table S1. Item-level reliability statistics for the seven-item medication access barrier score**

| **Item** | **Corrected item–total correlation** | **Cronbach’s alpha if item deleted** |
| --- | --- | --- |
| Difficulty obtaining medications or accessing healthcare facilities | 0.446 | 0.750 |
| Health services in the area were disrupted or stopped due to the war | 0.427 | 0.754 |
| Medications in the area were damaged due to power outages | 0.490 | 0.741 |
| Medications were not easily available in the area | 0.529 | 0.733 |
| Difficulty obtaining medications due to high prices | 0.516 | 0.736 |
| Difficulty accessing healthcare facilities due to shortages of transportation and fuel | 0.559 | 0.726 |
| Pharmacies outside Homs have medication shortages | 0.463 | 0.746 |

***Note.*** *Overall Cronbach’s alpha was 0.769 (N = 1,042). All corrected item–total correlations exceeded 0.30, and deletion of any item reduced the overall alpha, supporting retention of all seven items.*
